# Projected Losses of Ecosystem Services Incurred by Reserve Resources of Cultivated Land Development and Development Priority: A Case Study of Linzhou City in Henan Province, China

**DOI:** 10.3390/ijerph19116627

**Published:** 2022-05-29

**Authors:** Zhuoyi Zhou, Tian Chen, Ling Li, Xiuli Wang, Xinwei Feng, Jie Lu

**Affiliations:** 1 College of Resources and Environment, Henan Agricultural University, Zhengzhou 450002, China; zyzhouzh333@stu.henau.edu.cn (Z.Z.); chentian816@stu.henau.edu.cn (T.C.); liling@henau.edu.cn (L.L.); wangxiuli@henau.edu.cn (X.W.); xwfeng@henau.edu.cn (X.F.); 2 Henan Engineering Technology Research Center for Land Reclamation and Ecological Restoration, Zhengzhou 450002, China

**Keywords:** RRCL, losses of ecosystem services, development priority, pricing of ecosystem services, Linzhou City, China

## Abstract

The development of reserve resources of cultivated land (RRCL) is a vital way of supplementing cultivated land, and plays a crucial role in ensuring food security. However, if we blindly pursue the quantity of development while ignoring the conservation of the ecosystem, we are likely to waste land resources and destroy the ecological environment. Therefore, it is necessary to address the urgent issue of preventing ecological risks resulting from the development of RRCL and to enhance the actual effect of supplementing cultivated land. Taking Linzhou City in Henan Province as an example, this paper first assessed the tillability of RRCL and estimated the functionality of ecosystem services. Then it projected the losses of ecosystem services incurred by RRCL development, based on which it determined the development priority. The following conclusions were drawn: (1) The total area of RRCL in Linzhou City amounts to 8845 hectares. (2) According to the research forecast, the total annual losses of ecosystem services incurred by RRCL development in Linzhou City include: water conservation of 776,200 m^3^, soil retention of 340.84 t, and carbon sequestration and oxygen release of 2311.12 t. Moreover, the total value of losses amounts to RMB 15.7754 million. (3) The ecological losses incurred by RRCL development vary with the different types of land. Overall, the ecological loss derived from reclaimable land is inferior to that of cultivable land, with the average value of ecological loss amounting to RMB 600 and RMB 5300 per hectare, respectively. The ecological loss from the development of class II land is: pond < garden land < artificial grassland < artificial forest land < natural grassland < bare land. Moreover, land development should be postponed when the quantity of ecological losses reaches level III or higher. (4) Corrections are made based on the ecological coefficient of the economic potential of RRCL development so as to determine the priority of development. The research findings indicate that the priority of development of eastern towns is higher than that of central and western towns in Linzhou City, with Donggang and Hengshui topping the priority list and Shibanyan, Lingyang, and Chengguan having the lowest priority.

## 1. Introduction

To alleviate the contradiction between the occupation of cultivated land by urban construction and the retention of cultivated land, the Ministry of Land and Resources rolled out the policy of cultivated land balance (CLB) in 1998. Local government also took measures to enhance the development and management of RRCL. RRCL is defined as land resources that can be directly applied into agricultural production only after development, reclamation, and consolidation, and can be divided into cultivable land and reclaimable land. As a primary source of supplementing cultivated land [1,2,3,4], RRCL plays a crucial role in maintaining the balance of cultivated land resources [5,6,7]. Over the recent years, thanks to the campaign of poverty alleviation and the implementation of the rural revitalization strategy, the agricultural structure has experienced accelerated transition, and the cultivated land is encountered with the severe challenge of recessive reduction [8], thus increasing the demand for supplementing cultivated land and heightening the pressure of RRCL shortage. During the policy implementation, some regional governments attach greater importance to the quantity than the quality of development and neglect the ecological function of RRCL [9,10]. Develop land that is not suitable for cultivation or has important ecological functions and is ecologically fragile as cultivated land. As a result, the regional ecological environment is devastated, and the funds of development are also wasted, thus severely undermining the sustainable use of land resources.

In view of the huge demand for supplementing cultivated land across China, numerous studies have been carried out on RRCL since the late 20th century, which primarily focus on the surveys and assessment of RRCL tillability [11,12,13,14,15] at the regional, provincial, and municipal levels based on natural conditions. To cope with the issues incurred by RRCL development, some scholars have analyzed the features of distribution [16,17,18] and the combination of development [19,20,21] of RRCL at the spatial scale, and others have studied the potential [22,23,24] and coping measures [25] related to RRCL development. As the development of new cultivated land shifts to the northern parts of China and arid areas [26], scholars have started to pay closer attention to the impact imposed by the carrying capacity of regional resources [27,28] on RRCL development. To tackle the ecological issues frequently caused by RRCL development, scholars have identified utterly effective coping measures from the aspects of ecological sensitivity [29] and ecological security [30]. The timing of development [17,31] is determined mainly based on such factors as the concentration of resources and the convenience of development.

To sum up, the majority of the existing studies on RRCL focus on achieving the quantitative target of supplementing cultivated land from the aspects of tillability assessment, potential evaluation, and spatial combination. With the increasing development of RRCL, numerous ecological issues have arisen from people’s insufficient awareness of potential ecological risks, inaccurate identification of ecological suitability, and deficiencies with respect to ecological assessment while determining the priority of development. Additionally, the newly developed cultivated land oftentimes features weaker production capacity of grains. Hence, the mode of RRCL development needs to be urgently modified.

Taking Linzhou City as an example, this study first assessed the tillability of RRCL and estimated the functionality of ecosystem services via the InVEST model and the Universal Soil Loss Equation (USLE) approach. Second, the study provided a forecast on the ecological loss from RRCL development based on the discrepancy of functionality between the ecosystem services of cultivated land and RRCL. Third, the study identified the value of ecological loss via the replacement price method and the shadow price method. Finally, it adjusted the ecological coefficient of the economic potential of RRCL development to determine the development priority so as to avoid the ecological risks of RRCL development and improve the quality of supplementing cultivated land. On the premise of attaining the goal of supplementing cultivated land, this study aims to enhance the system of ecological management for RRCL development, thus laying a solid foundation for the scientific demarcation of RRCL during territorial spatial planning.

## 2. Overview of the Research Area and Statistical Sources

### 2.1. Overview of the Research Area

**Figure 1:**
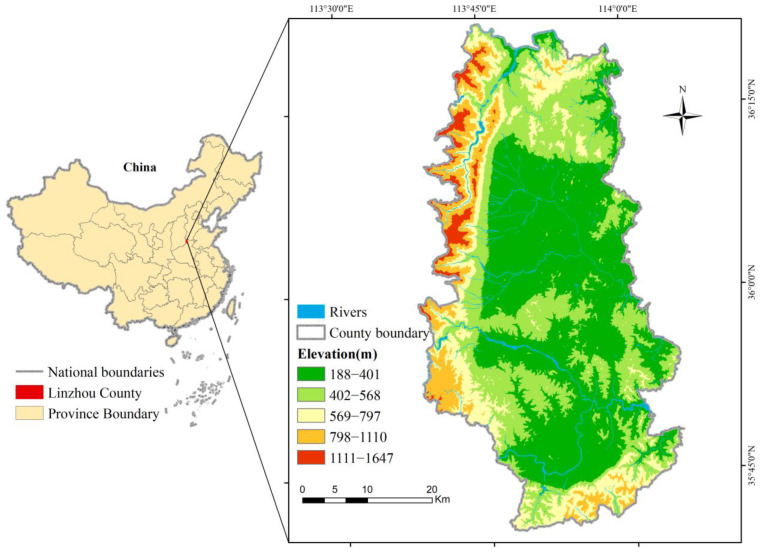
Location and elevation of Linzhou City.

Linzhou City is located in the northern parts of Henan Province, at the junction of Henan, Shanxi, and Hebei Provinces. The city is situated at the eastern foot of the Taihang Mountains, with geographical coordinates of 113°37′, 114°51′ E and 35°40′, 36°21′ N. Covering an area of 2046 km^2^, Linzhou City features the jurisdiction over 17 towns, including Donggang, Hengshui, and Heshun. In 2020, the city’s GDP amounted to RMB 56 billion with the registered population reaching 1.14 million people and the per capita disposable income reaching RMB 28,086. Linzhou City features numerous hills but few plains, with slopes and hills accounting for 86% of the total area. It has low altitude in the middle and eastern parts of the city and higher altitudes in the south, north, and west. The city features a temperate monsoon climate, with four distinct seasons. Moreover, the majority of rainfalls take place in summer, with annual precipitation reaching 600–700 mm. With the abundance of hills and slopes, the city has richer RRCL resources compared with other parts of Henan Province. The location of Linzhou City is shown in Figure 1.

### 2.2. Statistical Sources and Data Processing

**Figure 2:**
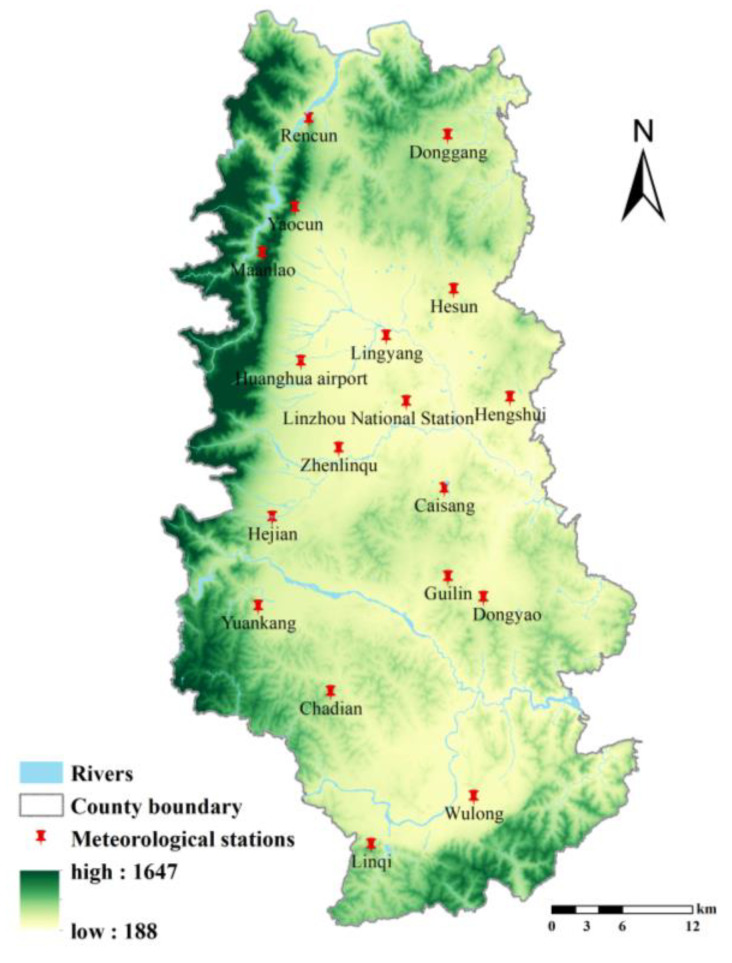
Distribution of meteorological stations in Linzhou City.

The data required in this study include the data on land use, soil, meteorology, landform, and vegetation cover. The specific types and sources of data are specified in Table 1. To obtain data with higher accuracy, we compared the spatial and temporal resolution of data from varying sources and eventually selected the data on vegetation coverage extracted on a monthly basis from the Landsat 8 Satellite using Google Earth Engine. Such data have a spatial resolution of 30 m and a temporal resolution of 16 d. With respect to the NDVI data extracted from the MOD13A1 dataset based on the MODIS satellite, it is more applicable to the accounting of county-scale ecosystem services. In addition, we selected the meteorological data monitored on a daily basis extracted from 18 meteorological stations in Linzhou City. The distribution of meteorological stations is illustrated in Figure 2.

With respect to the processing of data on RRCL tillability assessment, first, the map spots of the types of land that can be used as RRCL were extracted from the data on land use. Subsequently, the factors of slope and elevation were extracted from DEM data, and the factors of soil texture and limiting of tillage layers were extracted from soil data, whereas the factors of annual precipitation and annual accumulated temperature were extracted from meteorological data. Last but not least, the extracted map spots of the types of land were superimposed with each factor, and the factor of tillage evaluation was added to the attribute table of map spots of the types of land in the form of fields. 

With respect to the processing of data on functionality accounting of ecosystem services, we converted vector data (date on land use) into raster data. The meteorological data are converted into raster data by spatial interpolation (kriging) according to the geographic coordinates of the site. Raster data (soil data, evapotranspiration data, and solar radiation data) with a spatial resolution other than 30 m were resampled to 30 m resolution. All raster data are adjusted to the same coordinate system for each model of ecosystem service estimation. The standards of pricing the loss value of ecological services originate from the Specifications for Assessing the Functionality of Forest Ecosystem Services in China [32], Budget Quota of Water Conservancy Construction Project of the Ministry of Water Resources of the PRC, and the agricultural information platform of the Ministry of Agriculture and Rural Affairs of the PRC.

## 3. Research Methods

### 3.1. Assessment of RRCL Tillability

Based on the varying methods of development and utilization, RRCL can be divided into cultivable land and reclaimable land. The cultivable land refers to the natural land previously subject to no human labor input and can be converted into cultivated land through land development. The reclaimable land refers to the damaged land and the artificial forest land and garden land that either are poorly developed and utilized or can be converted into cultivated land through industrial restructuring. For the purpose of clarifying the research findings and ensuring that these findings are more applicable to the land management, according to the Land Classification of the Third National Land Resource Survey (GB/T21010-2017), the RRCL was divided into six categories: garden land, artificial forest land, natural grassland, artificial grassland, bare land, and pond (Table 2).

In terms of the extraction of the reserve resources of reclaimable land, based on the research needs, the adjustable or reclaimed land was deemed reclaimable land in the study. The definition of RRCL was clarified during the Third National Land Resource Survey, and annotations of spot attributes were added to the present types of land [33]. Specifically, attributes were added for the adjustable land type based on the current use, including “recovery enabled by engineering projects” and “recovery to promptly take place”. According to the findings of the Third National Land Resource Survey conducted in Linzhou City, the spots of adjustable land types with planting attributes were extracted and marked as “recovery enabled by engineering projects” and “recovery to promptly take place”. The map spots within the ecological red line or the boundary of urban development were unsuitable for use as cultivated land since they must meet the requirements of ecological conservation and urban development, and thus, they were not used as RRCL. The rest of the land was deemed reserve resources of reclaimable cultivated land.

With respect to the assessment of tillability of the cultivable RRCL, according to provisions on the types of reserve resources of cultivated land stipulated in the Technical Regulations for Investigation and Assessment of RRCL [34], such land as other grassland, saline–alkali land, sandy land, and bare land are extracted as RRCL based on the findings of the Third National Land Resource Survey conducted in Linzhou City. Subsequent to the holistic analysis of the natural, ecological, and social conditions required for RRCL development (as specified in Table 3), as the most fundamental conditions for the development of RRCL, the evaluation index and criteria for natural conditions were identified based on the provisions in the Technical Regulations for Investigation and Assessment of RRCL [34] and the Technical Specification for Comprehensive Control of Soil and Water Conservation—Technique for Erosion Control of Slope Land [35] and Related Research Findings [12,23]. Under circumstances when the accumulated temperature is <1800 ℃, annual precipitation is <400 mm, irrigation conditions are absent, and elevation is >1500 m, the hydrothermal conditions of crop growth cannot be met, thus leading to the declines of crop yield. Land reclamation with a slope of >25° risks exacerbating the losses of surface soil. Optimal cropping conditions are necessary to facilitate crop growth, and high gravel content and tillage layer barriers will hinder the growth of crop roots, leading to the incapacity of supplying sufficient nutrients.

According to the principle of assessment method of limiting factor, all items of the system of indicators are regarded as units of assessment so as to identify the tillable reserve resources, and the rest are deemed untillable reserve resources. When the results of the assessment are part tillable and part untillable for the same map spot, we will take the assessment result of the area of over 50% as the final result, and the map spots are not divided.

### 3.2. Estimation of Ecological Regulation Services of Reserve Resources

Ecosystem services refer to the varying benefits that humans obtain from ecosystems [36,37], which are primarily divided into four categories [38,39], namely, supply service, regulation service, cultural service, and support service. When the type of ecosystem changes before and after RRCL development, the ecosystem services that it provides are also subject to changes. It is one-sided to measure the ecological suitability of RRCL development solely from the strengths and drawbacks of natural conditions, which is not a method of effectively preventing and controlling the potential ecological risks. Integrating the loss of ecosystem services into the development assessment system provides an accurate basis for measuring the ecological suitability of RRCL development.

This study estimates the services of ecosystem regulation derived from RRCL development from the aspect of function value [40,41,42]. Based on relevant studies, the services of wind breaking and sand fixation provided by China’s terrestrial ecosystem are concentrated in western regions [43] but are scarcer in central and eastern regions. Therefore, only the services of water conservation, carbon sequestration, and oxygen release as well as soil retention provided by RRCL are measured in Linzhou City. 

#### 3.2.1. Estimation of Water Conservation Services

Although a clear definition is yet to be given for the water conservation [44], a widely recognized concept in a narrow sense is provided as follows: Water conservation refers to the transition of surface water into surface runoff or groundwater through interception, absorption, and storage of precipitation by vegetation canopy and soil layer of varying categories of land use [45]. Based on the principle of water equilibrium, the difference between water input and output in a certain period was deemed water conservation in this paper. During the measurement of the water conservation of RRCL, the water yield module is adopted in the Integrated Valuation of Ecosystem Services and Trade-offs (InVEST) model [46] jointly developed by Stanford University, the World Wide Fund for Nature (WWF), and the Nature Conservancy (TNC). Thanks to its optimal simulation effects, the aforementioned InVEST model is the most widely used model and is also extensively adopted in numerous studies on water conservation in China [47,48,49,50]. The equation for estimating the water yield module is specified as follows:

(1) $$Y_{xj}=\left(1-\frac{AET_{xj}}{P_{x}}\right)\times P_{x}$$

(2) $$\frac{AET_{xj}}{P_{x}}=\frac{1+W_{x}R_{xj}}{1+W_{x}R_{xj}+\frac{1}{R_{xj}}}$$

(3) $$w_{x}=Z\frac{AWC_{x}}{P_{x}}$$

(4) $$R_{xj}=\frac{K_{xj}\times ET_{0}}{P_{x}}$$

where $Y_{xj}$ refers to the annual water production of raster cell x for class j land cover; $AET_{xj}$ refers to the actual evapotranspiration of class j land cover in raster cell x; *Px* refers to the precipitation of raster cell x; $W_{x}$ refers to the nonphysical parameter of the natural climate–soil properties; $R_{xj}$ refers to the Budyko drying index (i.e., the ratio of actual evapotranspiration to precipitation); *Z* refers to the Zhang coefficient [51]; $AWC_{x}$ refers to the effective soil water content of raster cell x, which is determined by the soil depth and physicochemical properties while referring to the findings of Zhou et al. [52]; $K_{xj}$ refers to the vegetation evapotranspiration coefficient of class j land cover in raster cell x; and *ET*_0_ refers to the reference crop evapotranspiration.

The biophysical codes of varying categories of vegetation are specified in Table 4, which are in essence the evapotranspiration coefficient and maximum root depth of varying types of vegetation. The table is configured while referring to the recommended parameters of the InVEST model [46] and related studies [47,49,53]. The table is specified as follows: 

#### 3.2.2. Estimation of Carbon Sequestration and Oxygen Release 

Carbon sequestration and oxygen release refers to the function of the vegetation of varying types of land use that could fix carbon and release oxygen [54]. The capacity of RRCL in carbon sequestration and oxygen release differs from that of cultivated land due to the distinct status of land cover and human interference. The capacity of an ecosystem in carbon sequestration and oxygen release is assessed by the annual net primary productivity (NPP) of varying types of vegetation, and the annual NPP of different categories of vegetation is measured with the model of solar energy utilization. Specifically, the NPP was calculated through the CASA model provided by Zhu Wenquan [55]. As a model of light energy utilization for estimating the NPP of vegetation, the CASA model has its unique advantages in that the required vegetation parameters can be obtained by remote sensing and applied to various regional scales. The NPP of the research area was estimated through the plugin in the ENVI model (i.e., Environment for Visualizing Images) developed by Zhu Wenquan et al. The equation of estimation is specified as follows:

(5) NPP(x,t)=APAR(x,t)×ε(x,t)

where NPP(x,t) is the NPP of vegetation of raster cell x in month t, *APAR*(*x*, *t*) refers to the photosynthetically active radiation absorbed by raster cell x in month t (unit: MJ/m^2^/month), and *ε*(*x*, *t*) refers to the actual utilization ratio of solar energy of raster cell x in month t (Unit: gC/MJ).

(6) APAR(x,t)=SOL(x,t)×FPAR(x,t)×0.5

where SOL(x,t) is the aggregate solar radiation of raster cell in month t (MJ/m^2^/month); *FPAR*(*x*, *t*) is the proportion of incident photosynthetically active radiation absorbed by vegetation in month t, and 0.5 is the ratio of the photosynthetically active radiation absorbed by vegetation to the aggregate solar radiation.

(7) $$\varepsilon \left(x,t\right)=T_{\varepsilon 1}\left(x,t\right)\times T_{\varepsilon 2}\left(x,t\right)\times W_{\varepsilon }\left(x,t\right)\times \varepsilon_{max}$$

where $T_{\varepsilon 1}\left(x,t\right)\;\mathrm{and}\;T_{\varepsilon 2}\left(x,t\right)$ are the limiting effects imposed by low temperature and high temperature on the light energy utilization rate, respectively; $W_{\varepsilon }\left(x,t\right)$ is the limiting effect imposed by the water state on the efficiency of light energy utilization; and $\varepsilon_{max}$ is the ideal state for the maximum light energy utilization (gC/MJ).

To facilitate the operation of the model, it is required to import the data on the types of land cover, monthly synthetic NDVI, precipitation, solar radiation, and mean temperature multiband data (raster). The simulated value derived from the model is referred for static parameters [56], the maximum efficiency *ε_max_* of solar energy utilization, and 95% NDVI (SRmax) and 5% NDVI (SRmin) of different types of land cover are provided. In addition, both NDVImax and NDVImin are configured based on the actual coverage of varying types of land cover.

#### 3.2.3. Estimation of Soil Retention

Soil retention refers to the capacity of preventing and controlling the erosion of the ecosystem and enhancing the storage and retention of water of sediments [57]. In this paper, the difference between potential and actual soil losses was taken as the estimated capacity of soil retention for different types of land cover based on the universal soil loss equation (USLE). This method features simpler parameters and easier calculation. It is capable of quickly simulating the spatial distribution features of soil conservation services during the holistic evaluation of various ecosystem services. Due to the disturbance of the soil environment by RRCL development, the surface soil tends to be looser and is prone to be eroded by running water. In addition, the transformation of slopes into terraces and the leveling of land will slow down the loss of soil. Hence, it is necessary to calculate the change in soil retention caused by RRCL. During the estimation of soil retention, the universal equation of soil loss is adopted, and the difference between potential and actual soil losses is regarded as the effects of soil retention for varying types of land cover. The equation is specified as follows:

(8) $$Q_{s}=Q_{pot}-Q_{act}$$

(9) $$Q_{pot}=R\times K\times L\times S$$

(10) $$Q_{act}=R\times K\times L\times S\times C\times P$$

where *Qs* refers to the soil retention for varying categories of land cover (t/km^2^·a); $Q_{pot}$ refers to the annual potential soil loss for a specific type of land cover; $Q_{act}$ refers to the actual soil loss; *R* refers to the rainfall erosion factor; *K* refers to the soil erosion factor; *L* refers to the slope length factor; *S* refers to the slope factor; *C* refers to the vegetation cover factor, and the *C* of varying types of land cover is obtained by referring to related studies [58]; and *P* refers to the soil retention measure factor.

### 3.3. Prediction of Ecological Loss Incurred by RRCL Development

#### 3.3.1. Prediction of Physical Loss of Ecosystem Services from RRCL Development

Without considering the temporary loss of ecosystem services incurred by RRCL development. In this paper, we took the average annual difference of ecological services derived from the utilization of cultivated land and the maintenance of reserve resources as the predicted value of the ecological service loss incurred by RRCL development:

(11) $$Q_{i}=\sum X_{j}(q_{ij}-G_{i})$$

where $Q_{i}$ refers to the loss of ecosystem regulation services of item i of RRCL, $X_{j}$ refers to the area of type j reserve resources, $q_{ij}$ refers to the ecosystem services of item i of type j RRCL per unit area, and $G_{i}$ refers to the ecosystem services of item i of cultivated land per unit area.

#### 3.3.2. Prediction of Value Loss of Ecosystem Services Incurred by RRCL Development

During the accounting of the value loss of ecosystem services, the cost of human and material resources required to recover or maintain equivalent ecosystem services was taken as the economic value loss through the replacement alternative cost method, shadow project method, market value method, and restoration cost method. The value loss of ecosystem services incurred by RRCL development includes several aspects, namely, water conservation, soil retention, and carbon sequestration and oxygen release. The equation is specified as follows:

(12) $$P_{g}=P_{w}+P_{s}+P_{c}$$

where $P_{g}$ refers to the value loss of ecosystem services incurred by RRCL development, $P_{w}$ refers to the value loss of water conservation, $P_{s}$ refers to the value loss of soil retention, and $P_{c}$ refers to the value loss of carbon sequestration and oxygen release.

During the accounting of the economic value of water conservation, the shadow project method is adopted. In other words, the cost of building and maintaining a reservoir of the same capacity is used to estimate the loss value of water storage and production in the ecosystem from RRCL development. The equation is specified as follows:

(13) $$P_{w}=C_{w}\times Q_{w}$$

where $P_{w}$ refers to the annual economic value loss of water conservation (yuan); $C_{w}$ refers to the cost of construction and maintenance of the unit capacity of reservoir (yuan/m^3^), and the $C_{w}$ of reservoirs in China is 6.11 (yuan/m^3^) according to the specifications for assessment of forest ecosystem services in China [32] set by the State Forestry Administration in 2008, which is equivalent to 15.4 (yuan/m^3^) in 2020; and $Q_{w}$ refers to the loss of annual water conservation incurred by RRCL development (m^3^).

The loss of the economic value of soil retention is measured from the aspects of soil nutrient loss and increased sediment deposition [44,59]. The value loss of soil retention incurred by RRCL development is assessed with the cost of increasing the same amount of soil nutrients and reducing sediment deposition. The equation is specified as follows:

(14) $$P_{s}=P_{s1}+P_{s2}$$

where $P_{s}$ refers to the economic value loss of soil retention, $P_{s1}$ refers to the value loss of soil nutrients (yuan), and $P_{s2}$ refers to the value loss of reducing sediment deposition (yuan).

Soil loss not only leads to the vanishing of soil particles, but is also attributable to the entrance of a large amount of soil nutrients into the water body along with the current, thus exacerbating the conditions of regional soil nutrients. The loss of soil nutrients (nitrogen, phosphorus, potassium, and soil organic matter) incurred by RRCL development is replaced with the price required to supplement the same amount of soil nutrients. The average contents of nitrogen, phosphorus, potassium, and organic matter in the soil of Linzhou City are estimated based on existing studies [60], namely, N: 1.27 g/kg, P: 22 mg/kg, K: 128 mg/kg, and organic matter: 23 g/kg. In addition, the contents of N, P, and K in diammonium phosphate and potassium chloride are assessed to reach 14%, 15.01%, and 50%, respectively. Based on the agricultural information platform of the Ministry of Agriculture and Rural Affairs of the PRC, the average producer prices of diammonium phosphate, potassium chloride, and biological organic fertilizer were estimated to be 2227 (yuan/t), 1958 (yuan/t), and 800 (yuan/t) in 2020, respectively. The equation for calculating the value loss of soil nutrients is specified as follows:

(15) $$P_{s1}=\sum \frac{\left(R_{i}\times Q_{s}\right)}{n_{i}}\times p_{i}$$

where $R_{i}$ refers to the average contents of total nitrogen, rapidly available phosphorus, and rapidly available potassium in soil; $Q_{s}$ refers to the loss of soil retention (t); $n_{i}$ refers to the contents of nitrogen, phosphorus, and potassium in the fertilizer, respectively (%); and $p_{i}$ refers to the unit price of the corresponding fertilizer (yuan).

Subsequent to entrance into the water system, the lost soil will lead to elevation of the riverbed and reduction of the reservoir capacity. Based on relevant studies, about 24% of the lost soil is deposited in river channels and reservoirs [36]. When the shadow project method is adopted, the dredging cost of a reservoir of the same capacity is used to assess the value loss of soil retention. The equation for assessing the value loss of reducing sediment deposition is specified as follows:

(16) $$P_{s2}=0.24\times \frac{Q_{s}}{\rho }\times c_{s}$$

where $Q_{s}$ refers to the loss of soil retention (t); ρ refers to the volume weight of soil (t/m^3^), and the ρ of surface soil in Henan Province refers to 1.34 t/m^3^ [61]; and $c_{s}$ refers to the unit dredging cost of a reservoir, and the $c_{s}$ was 12.6 yuan/m^3^ in 2002 according to the Budget Quota of Water Conservancy Construction Project of the Ministry of Water Resources of the PRC, which is equivalent to 46.6 yuan/m^3^ in 2020.

The value loss of carbon sequestration and oxygen release is divided into two parts, namely, the value loss of carbon sequestration and that of oxygen release. The value loss can reflect the market price of annual loss of carbon sequestration and oxygen release in the ecosystem from RRCL development. The equation is specified as follows:

(17) $$P_{c}=P_{c1}+P_{c2}$$

where $P_{c}$ is the value of losses of economic value of carbon sequestration and oxygen release,$\;P_{c1}$ is the value of losses of carbon sequestration (yuan), and $P_{c2}$ is the value of losses of oxygen release (yuan).

The value loss of carbon sequestration is estimated based on the carbon tax law, and the levy ratio is USD 150/t of CO_2_ equivalent referring to universal Swedish carbon tax in environmental economy [62,63,64], with the average exchange rate of USD/RMB of 6.89 in 2020. The equation is specified as follows:

(18) $$P_{c1}=1.63\times Q_{c}\times C_{C}\times 27.27\%$$

where $P_{c1}$ refers to the value loss of carbon sequestration (yuan); t refers to the amount of fixed carbon dioxide for 1 kg of organic matter produced by plants: 1.63 kg; $Q_{c}$ refers to the value loss of carbon sequestration and oxygen release NPP (t); and $C_{C}$ refers to the price of carbon dioxide (yuan/t).

The loss of oxygen release is estimated with the following equation:

(19) $$P_{c2}=Q_{c}\times 1.19\times O_{c}$$

where $P_{c2}$ refers to the value loss of oxygen release (yuan); $Q_{c}$ refers to the value loss of carbon sequestration and oxygen release NPP (t); $O_{c}$ refers to the price of producing oxygen (yuan/t), which is consistent with the specifications for assessment of forest ecosystem services in China [32] set by the State Forestry Administration in 2008.

### 3.4. Determination of the Priority of RRCL Development Based on Ecological Profit–Loss

Proper priority of RRCL development can facilitate the reduction of the interference to the ecosystem and the loss of ecosystem services. At present, the timing of RRCL development is primarily determined based on natural conditions [31] or the input–output ratio [17] without taking into account ecological factors. This study has taken the ecological loss incurred by RRCL development as a crucial factor to determine the priority of RRCL development. Subsequent to the nondimensionalization of the loss of three sorts of ecosystem regulation services and analysis with ArcGIS superposition, the spatial distribution can be identified for the loss of ecosystem regulation services of RRCL. Then, the priority of RRCL development of varying types of land can be determined accordingly. During the determination of the priority of RRCL development, we should not only regard the quantity as the decisive factor. For the purpose of preventing the ecological risks caused by development, the ecological coefficient of the economic potential of RRCL development should be adjusted accordingly. Given that the natural conditions of RRCL in Linzhou City share similar features, there is limited difference in the input–output ratio of development. Therefore, only the area of RRCL is adopted as the development potential. By taking the logarithm of the ratio of the RRCL with a low level of regional ecological loss to the total number as the coefficient of ecological correction, we adjusted the ecological coefficient of the economic potential of RRCL development, thereby obtaining the development priority. The model of development priority is specified as follows:

(20) $$Y_{i}=Z_{i}\times E_{i}$$

where $Y_{i}$ refers to the priority of RRCL development in each town, $Z_{i}$ refers to the development potential of RRCL in each town ($Z_{i}$ = area of RRCL in each town/total area of RRCL in Linzhou City), and $E_{i}$ refers to the ecological coefficient (EXP (area of class I and II RRCL in each town/total area of RRCL in Linzhou City)).

## 4. Analysis of Results

### 4.1. Assessment Results of RRCL Tillability 

**Figure 3:**
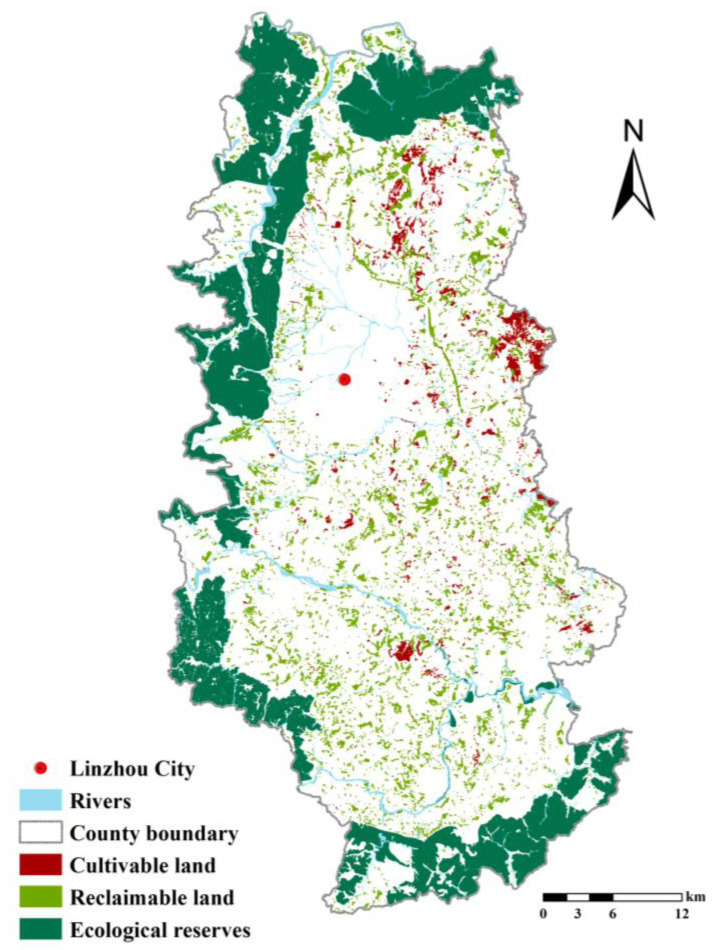
Tillable RRCL located in Linzhou City.

The waste grassland, saline–alkali land, sandy land, and bare land in Linzhou City were evaluated from the aspect of tillability in accordance with the established system of evaluation index of RRCL tillability. The assessment results indicate that in Linzhou City, there are 2233 hectares of cultivable RRCL in total, among which, 2120 hectares are grassland (95%), and the rest are bare land. Moreover, there are 6612.8 hectares of reclaimable RRCL, including artificial forest, grassland, garden, and pond, whose recovery is either enabled by engineering projects or to promptly take place. The area of varying sorts of RRCL in Linzhou City is specified in Table 5, and the area of RRCL in each town of Linzhou City is specified in Table 6. The distribution of cultivable and reclaimable RRCL in Linzhou City is illustrated in Figure 3.

The cultivable RRCL are primarily distributed in hilly and gently sloping areas in the middle and eastern parts of Linzhou City. With respect to the influencing range of assessment indicators, the major limiting factors of RRCL development in Linzhou City include the thin plough layer and the high gravel content. There is rocky land with a thin soil layer in the hilly area of Linzhou City due to the geological structure of the Taihang Mountains with limestone development.

### 4.2. Estimation Results of Physical Calculation of Ecosystem Regulation Services

**Figure 4:**
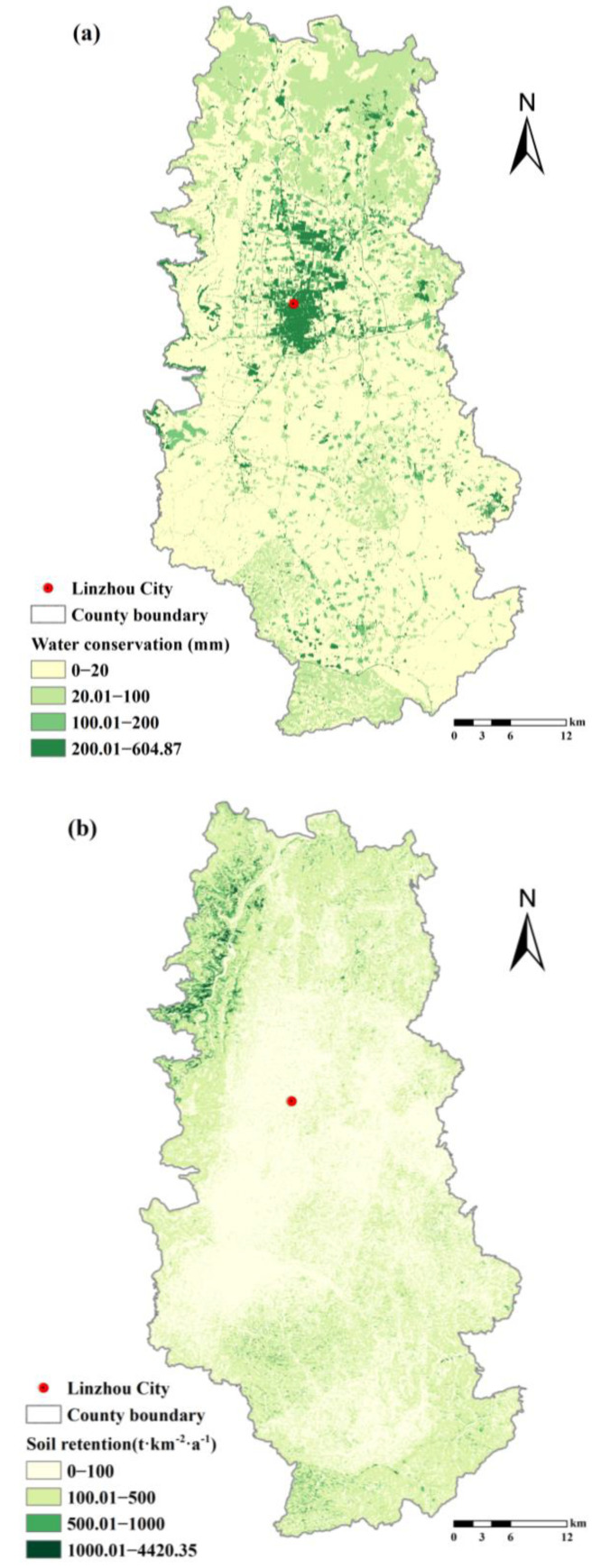

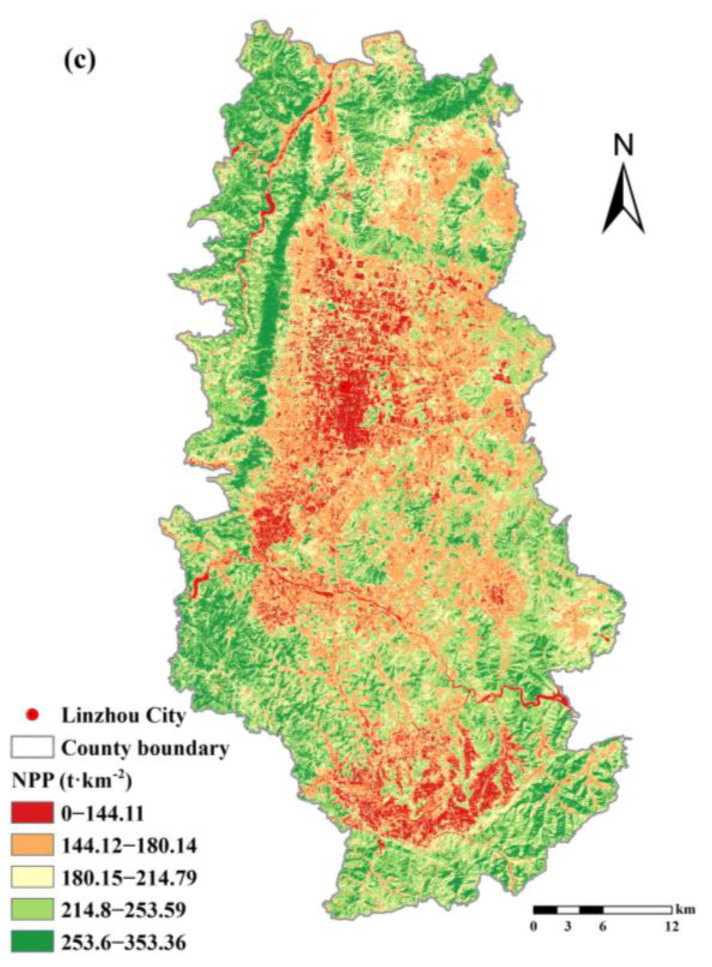
Physical quantity of ecosystem regulation services in Linzhou City: (**a**) water conservation, (**b**) soil retention, and (**c**) carbon sequestration and oxygen release.

After calculation, the quantity of the regulating services of the ecosystem in Linzhou City was obtained (Figure 4). In terms of water conservation (Figure 4a), artificial construction land > shrub > grassland > forest > cultivated land > water body, which is derived from the discrepancy in terms of the runoff producing capacity and evapotranspiration capacity of varying types of land cover. In terms of soil retention (Figure 4b), artificial forest and grassland feature a larger capacity, while water bodies feature a smaller capacity. From the spatial aspect, mountainous and hilly areas feature a larger capacity, while plains and valley areas feature a smaller capacity. This is derived from the discrepancy in terms of the potential soil loss of the soil surface of varying slopes and soil properties, and is attributable to the difference of the vegetation coverage of varying types of land cover. In terms of carbon sequestration and oxygen release NPP (Figure 4c), natural forest, shrub, and grassland feature a larger capacity, while cultivated land, artificial construction, land and water bodies feature a smaller capacity.

Based on the calculation of regulating services of Linzhou City, the value of water conservation, soil retention as well as carbon fixation and oxygen release of cultivated land and RRCL were extracted according to the location. Overall, with respect to the average water conservation (mm), cultivable land > reclaimable land > cultivated land. With respect to the average soil retention (t·km^−2^·a^−1^), cultivable land > reclaimable land > cultivated land. From the aspect of the types of ecosystems, cultivated land and reclaimable RRCL are categorized as artificial ecosystem or seminatural ecosystem, and cultivable RRCL is categorized as natural ecosystem. Different types of ecosystems vary in their capacity of self-regulation, so there is discrepancy in the ecosystem services to be provided by these ecosystems. To cope with the issue of deficient ecological prevention and control in RRCL development, attention must be paid to such discrepancy and its potential consequences. The services of ecological regulation of RRCL of varying types of land are specified in Table 7.

### 4.3. Analysis of the Results of Predicted Loss of Ecosystem Services Incurred by RRCL Development 

#### 4.3.1. Analysis of the Results of the Predicted Physical Loss of Ecosystem Services Incurred by RRCL Development 

Judging from the estimation results of three functions of ecosystem services, RRCL had higher capacity than cultivated land in terms of the average supply of ecosystem regulation services per unit area. Therefore, the reclamation of RRCL for cultivated land will inevitably lead to the losses of ecosystem services. The average loss of ecosystem services per hectare was specified as follows: For developable cultivated land, there was water conservation of 324.3 m^3^, soil conservation of 0.08 t, and carbon fixation and oxygen release of 0.3 t; for reclaimable cultivated land, there was water conservation of 11.30 m^3^, soil conservation of 0.02 t, and carbon fixation and oxygen release of 0.25 t. In general, with respect to the loss of ecosystem services incurred by RRCL development, cultivable land > reclaimable land. From the perspective of various ecosystem services, grassland and bare land suffered from higher losses of water conservation, gardens suffered from higher losses of soil conservation, and grassland and artificial forests suffered from higher losses of carbon fixation and oxygen release. According to the assessment of this study, the ecological loss incurred by RRCL development does not include the loss of ecosystem services incurred by the disturbance of the building of supporting facilities during RRCL development. The loss of varying types of RRCL is specified in Table 8.

#### 4.3.2. Value of the Losses of Ecological Services Incurred by RRCL Development in Linzhou City

The value of losses of ecological services incurred by the RRCL development was estimated by referring to the loss of ecological services from RRCL development and the pricing standards of varying sorts of services (Table 9). The total value loss of ecosystem services incurred by the RRCL development in Linzhou City amounts to RMB 15.7754 million per year, including cultivable RRCL of RMB 11.9107 million and reclaimable RRCL of RMB 3.8647 million. In addition, the average ecological loss of cultivable RRCL amounts to CNY 5300 per hectare, and reclaimable RRCL amounts to CNY 600 per hectare. 

### 4.4. Priority of RRCL Development in Linzhou City

**Figure 5:**
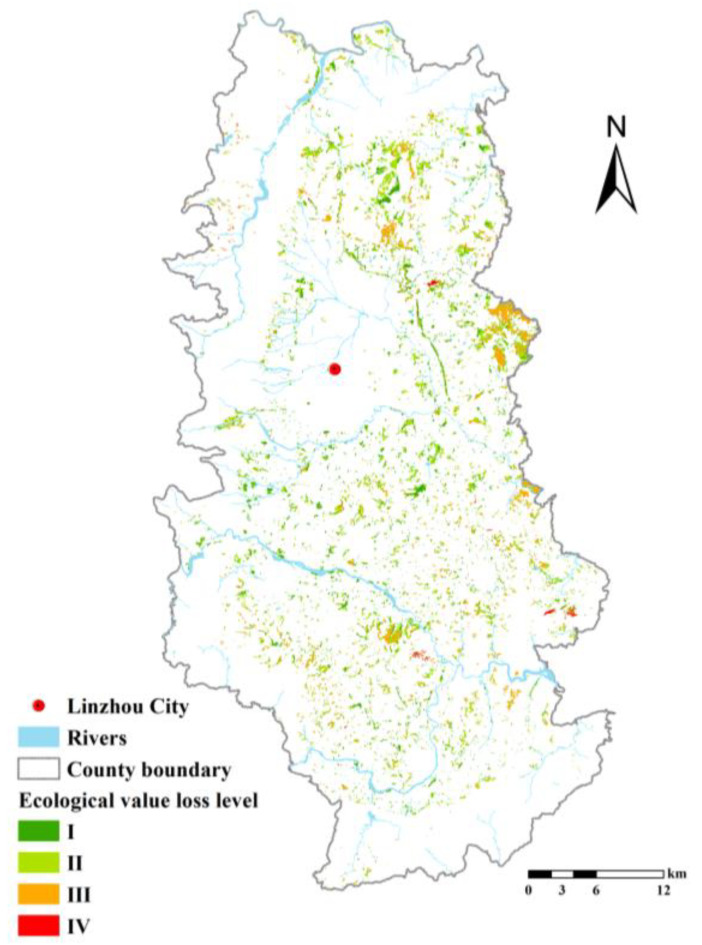
Levels of ecological loss of RRCL development.

The natural breaks method (Jenks) is adopted to divide the ecological loss of all types of RRCL in Linzhou City into four levels so as to identify the spatial distribution of all levels (Figure 5). A low level of ecological loss indicates minor loss of ecological services and higher priority of development. Level I indicates no loss or utterly low loss of value of ecosystem services, and priority can be given to the development accordingly. Level II indicates small loss of development loss. Moreover, level III indicates huge loss of development, and thus an ecologically friendly approach should be adopted in the development to minimize interference to the ecosystem, and the ecological slope and the network of farmland forests should be established to compensate for the loss of ecosystem services. Level IV indicates that the development risks leading to the deterioration of the ecological environment. From the perspective of balanced income, higher ecological costs can lead to lower benefits of land output, making it prone to the waste of land development funds. Hence, the development should be postponed. Within the determined level of ecological loss, priority should be given to the development of contiguous land that is easier to develop. 

Judging from the average value loss of ecological services incurred by varying types of RRCL development (Table 9), it can be seen that reclaimable land features smaller loss compared with cultivable land. As regards class II land, pond < garden < artificial grassland < artificial forest < natural grassland < bare land. Hence, the land should be developed in the decreasing order of loss.

**Figure 6:**
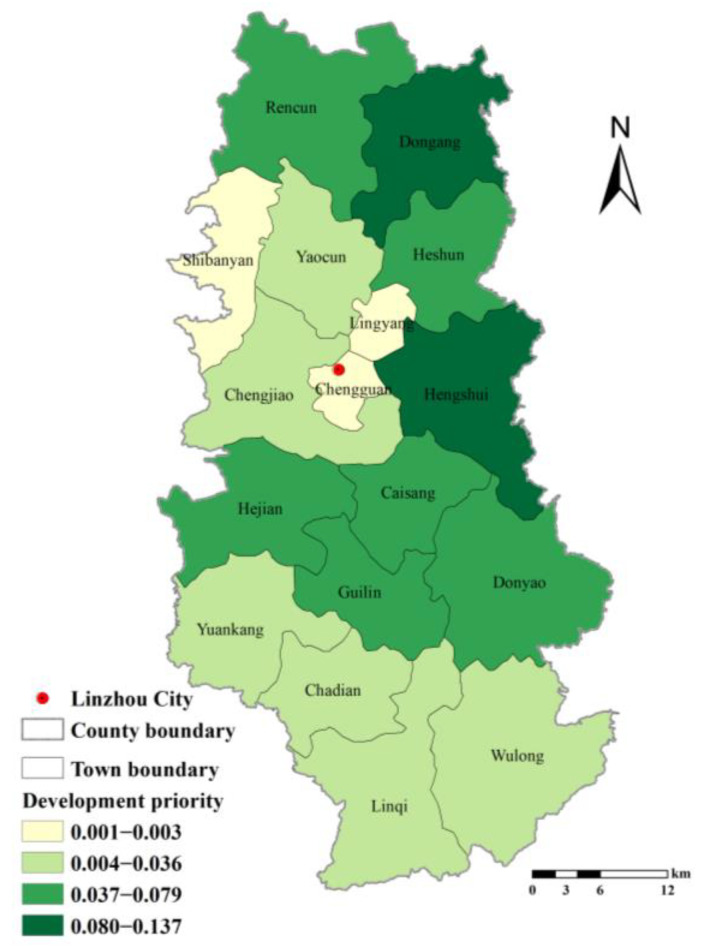
Priority of RRCL Development in Linzhou City.

The priority of the development of RRCL in Linzhou City is determined by correcting the ecological coefficient of the economic potential of RRCL development in each town and grading the priority of development (Figure 6). The results indicate that the priority of development is higher in eastern towns than in central and western towns, with Donggang and Hengshui having the highest priority and Shibanyan, Lingyang, and Chengguan having the lowest priority.

## 5. Discussions

The shrinkage of RRCL is not merely an issue faced by China, but a challenge taking place around the globe. Unreasonable development is speeding up this process and poses a huge threat to the ecological environment [65]. At present, the development of RRCL is still in the stage of meeting the quantitative goal of the index of cultivated land. Although the central government has repeatedly provided land that is unsuitable for farming or ecologically vulnerable, which shall not be reclaimed for farmland, the situation has yet to be essentially improved. In addition to the insufficient understanding of local governments, another crucial factor is that the ecological suitability of RRCL is defined vaguely, making it harder to apply the evaluation results. This paper provides a fresh concept for the ecological suitability evaluation of RRCL development. Ecological loss is used to estimate the ecological suitability and to convert ecological loss into a monetary form. Hence, it becomes easier to apply the evaluation result and to more accurately assess the appropriateness of local governments’ behavior during farmland development.

Looking forward, based on the identification of tillability of RRCL, we should enhance the mode of phased development based on the forecast on ecological profit–loss, and ensure that the new cultivated land can meet the requirements of grain production in three dimensions, namely, quantity, quality, and ecology. We shall extend the path of ensuring ecological protection and provide better guidance on renewing the ecologically friendly and efficient mode of RRCL development by facilitating the compensation of ecological loss. Local governments should abandon the highly subjective mode of decision making related to development for the sole purpose of matching the quantitative indicators [9], and the authority shall take tillability and ecological suitability as the basis to enhance the development and management of RRCL. From the aspect of food security, the ecological loss incurred by the development of reclaimable RRCL should be smaller than that incurred by the development of cultivable RRCL. It is more economical to enhance “nongrain” land than to reclaim new cultivated land. In addition, the production capacity of developing existing cultivated land is superior to that of reclaiming new cultivated land [66]. 

In terms of the estimation and pricing of ecosystem losses, this study selected three major functions of ecosystem regulation services and only measured the difference of ecosystem services of varying types of land use. With the constant expansion and deepening of the connotation of ecosystem services, the scope of ecosystem services can be extended based on the needs of future development. For instance, the accounting of windbreak and sand fixation can be added in the western areas, and the cultural value of landscape can be added in the surrounding area of natural landscapes [67]. With respect to the object of accounting, the RRCL development process and supporting facilities can also be included into the accounting system. Due to the constraints of the accuracy of data acquisition, the assessment results of ecosystem services are subject to the impact imposed by the average value of regional factors to a significant extent. In reality, the functional accounting approach should be adopted based on the measured value of numerous factors in the plot. The pricing of ecological loss should be replaced or converted based on the current market price of varying types of services [68], which can reflect the actual price of losses of ecosystem services. For those parts that are difficult to price in the ecological compensation of the development of reserve resources, it is necessary to explore the establishment of a compensation system based on the tax, deposit, and fund of ecological compensation. When it comes to the transaction of transregional quota of new cultivated land, remote ecological compensation shall be adopted so as to address the regional injustice and to transfer the dividends brought by the development of construction land. However, ecosystem services shall be deemed a flow asset rather than a stock asset. They are worth studying further in determining the period and selecting the methods of compensation [69].

Since the primary goal of developing cultivated land is to harvest agricultural products so as to obtain economic returns, it is of great significance for a country to ensure food security by cultivating a sufficient amount of cultivated land. Therefore, people often tend to solely pay attention to the economic potential of RRCL, while neglecting its ecological status. In previous studies, only a simple evaluation of ecological suitability was carried out prior to the development of RRCL, but sufficient ecological guidance was absent during the development of RRCL. This paper elaborates on a more reasonable development priority by adjusting the ecological coefficient of the economic potential of RRCL development, matching the development of RRCL with the quantitative requirements and minimizing the possibility of ecological risk.

In this paper, only the value of losses of ecosystem services incurred by the reclamation of RRCL is calculated for cultivated land. In fact, the value of losses of ecosystem services of converting natural land into construction land is much higher than that of converting natural land into cultivated land. Artificially transforming the types of land use will lead to changes of the types of ecosystems. Due to the discrepancy between different types of ecosystems, such transformation will inevitably incur losses of ecosystem services, which may heighten ecological risks. The method provided in this paper is not only applicable to the forecast of losses of ecosystem services incurred by RCL development in other regions, but also applicable to the forecast of losses of ecosystem services incurred by the transition into other types of land use, providing the basis for developing more ecologically friendly strategies for artificial conversion of land types. It should be pointed out that there are differences in the connotation of ecosystem regulation services for different land-use types, so the content of ecosystem service estimation should be adjusted to make the estimation results more reasonable.

## 6. Conclusions

This study takes Linzhou City of Henan Province as an example and focuses on the negligence issue of ecosystems during RRCL development from numerous aspects, including tillability assessment, ecosystem services and estimation of value loss, determination of the priority of development, and subsequent ecological compensation with the assessment of ecological profit–loss at the core. The study aims to provide a basis for forestalling ecological risks, preserving ecosystems, and enhancing the efficiency of RRCL development, and also provides a fresh concept for the scientific and reasonable identification of RRCL and the regulation of land-use zoning during the spatial planning of land. The following conclusions have been reached:

(1) First, this study established a system of assessment indicators of RRCL tillability based on natural, ecological, and social conditions to assess the tillability of grassland, saline–alkali land, sandy land, and bare land in Linzhou City so as to properly determine the scope of RRCL. Judging from the assessment results, there are 2233 hectares of tillable and reclaimable RRCL in total in Linzhou City, among which 2120 hectares are grassland (95%). With respect to the influencing range of assessment indicators, the major limiting factors of RRCL development in Linzhou City include the thin plough layer and the high gravel content. In addition, there are 6612.8 hectares of reclaimable RRCL, including artificial forest, grassland, garden, and pond, whose recovery is either enabled by engineering projects or to promptly take place. These results are obtained according to the Third National Land Resource Survey conducted in Linzhou City. Not all lands are suitable for development as cultivated land, and it is necessary to conduct a reasonable tillability evaluation before the development of RRCL. The purpose of this is to make the newly cultivated land have enough capacity to produce crops. Additionally, it can be profitable by obtaining enough economic remuneration to cover the cost of development, instead of making the development cost irrecoverable, resulting in a waste of development funds.
(2) The results of calculation and comparison of regulating services from the RRCL development in Linzhou City indicated that the average ecosystem services of RRCL exceeded the level of cultivated land; the average water conservation from cultivable, recoverable and cultivated land reached 39.5, 8.2 and 7.07 mm, respectively; the average soil retention reached 101.25, 95.3 and 93.3 t·km-2·a-1, respectively, and the average carbon fixation and oxygen release reached 201.6, 196.6 and 171.8 t·km-2, respectively. From this result, it can be seen that the ecosystem services of RRCL are higher than those of cultivated land. Additionally, the development of RRCL will cause the loss of ecosystem services. Therefore, it is necessary to predict the loss of ecosystem services in the development of RRCL.
(3) This study projected the total annual loss of ecosystem services incurred by RRCL development in Linzhou City based on the assessment of tillable RRCL and the estimation of ecosystem regulation services. The annual losses of ecosystem services for cultivable and reclaimable land included: water conservation of 701,600 and 74,600 m^3^, respectively; soil retention of 179.5 and 161.34 t, respectively; and carbon fixation and oxygen release of 667.4 and 1643.72 t, respectively. Each ecological service is priced based on the shadow project method and the market value method, and the total annual loss of ecosystem regulation services incurred by RRCL development in Linzhou City is measured to be RMB 15.7754 million. Specifically, the loss of cultivable reserve resources amounts to RMB 11.9107 million, that of reclaimable land amounts to RMB 3.8647 million, and the unit area losses of cultivable and reclaimable land amount to 0.53 and 0.06 (10,000 yuan/hm), respectively. The loss of ecosystem services per unit area is as follows: reclaimable land < cultivable land. Therefore, the development of reclaimable land has higher ecological suitability and lower ecosystem service losses. In fact, since a certain amount of human labor and capital has been invested in reclaimable land, the cost of developing reclaimable RRCL is lower than that of cultivable land and has a higher economic return.
(4) Proper priority of development is determined according the ecological loss of various sorts of RRCL. In terms of the average value loss of ecological services incurred by varying types of RRCL development, pond < garden < artificial grassland < artificial forest < natural grassland < bare land. Hence, various sorts of RRCL should be developed in the decreasing order of losses. The development priority of RRCL in Linzhou City is determined by adjusting the ecological coefficient of the economic potential of RRCL development in each town. The results indicated that development priority is higher in eastern towns than in central and western towns, with Donggang and Hengshui having the highest priority and Shibanyan, Lingyang, and Chengguan having the lowest priority. When only considering ecological suitability, it is reasonable that land types with low ecological loss levels have higher development priorities. However, the factors considered in actual development also include the development potential of RRCL. Therefore, this paper corrects the ecological coefficient of the development potential of RRCL. This will make up for the shortcoming of only focusing on the economic potential and ignoring ecological suitability in determining the priority of RRCL development. Additionally it will make the priority of RRCL development meet the economic potential and ecological suitability at the same time.

## Figures and Tables

**Table 1:** Types and sources of data.

Types of Data	Sources of Data	Format of Data
Data on the land use in 2020	Third National Land Resource Survey conducted in Linzhou City	Vector
DEM data	Resource and Environment Sciences and Data Center (http://www.resdc.cn, accessed on 25 May 2022)	Raster (30 m × 30 m)
Soil data	Scientific Data Center in Cold and Arid Regions (http://westdc.westgis.ac.cn/, accessed on 25 May 2022)	Raster (1 km × 1 km)
Data on China’s county-level administrative boundary in 2015	Resource and Environment Sciences and Data Center (http://www.resdc.cn, accessed on 25 May 2022)	Vector
NDVI data in 2020	TOA image collection from the Landsat 8 Satellite (https://earthengine.google.com, accessed on 25 May 2022)	Raster (30 m × 30 m)
Meteorological data of Linzhou City in 2020 (daily temperature, daily precipitation)	Linzhou Fengyun Meteorological Development Center	Table
Evapotranspiration data	Dataset on the Global Potential Evapotranspiration and Global Aridity Index (https://cgiarcsi.community/data/global-aridity-and-pet-database/, accessed on 25 May 2022)	Raster (90 m × 90 m)
Data on the red line of ecosystems and the boundary of urban development	Territorial and spatial planning of Anyang City	Vector
Date on the solar radiation	NASA MODIS (https://ladsweb.modaps.eosdis.nasa.gov/, accessed on 25 May 2022)	Raster (1 km × 1 km)

**Table 2:** Comparison sheet of RRCL.

Class I	Classification in the Study (Class II)	Classification in the Third National Land Resource Survey
Reclaimable land	Artificial forest	Shrub
		Arbor forest
		Other forests
	Garden	Orchard
		Other gardens
	Pond	Pond
		Breeding pond
	Artificial grassland	Artificial pasture
		Other grasslands
Cultivable land	Natural grassland	Waste grassland
		Other grasslands
	Bare land	Idle land
		Bare land
		Naked rocky and gravel land
		Sandy land

Note: Other grassland in the category of artificial grassland refers to the artificially planted grassland except for artificial pasture, and other grassland in the category of natural grassland refers to other sorts of natural grassland except for waste grassland.

**Table 3:** System of assessment indicators for the RRCL tillability of Linzhou City.

Criterion Layer	Assessment Indicator	Assessment Criterion	
		Tillable	Untillable
Natural condition	Annual temperature > 10 °C	>1800 °C	<1800 °C
	Annual precipitation	>400 mm	<400 mm and without irrigation conditions
	Slope	<25°	≥25°
	Elevation	<1500 m	≥1500 m
Plough layer	Soil texture	Clayey, loamy, sandy	Gravel content > 30%
	Plough layer obstacle	None	Thin soil body and shallow lithoidal texture
Ecological condition	Ecological effect	Exterior to the ecological red line	Within the ecological red line
Social condition	Retention for construction	Exterior to the boundary of urban development	Within the boundary of urban development

**Table 4:** Biophysical codes of the InVEST model.

Type of Land Use	Code of Land Use	Kc	Maximum Root Depth
Dryland	10	0.8	2200
Forest	20	1	3100
Grassland	30	0.6	2400
Shrub	40	0.6	2600
Wetland	50	1.2	100
Water surface	60	1	1
Rural settlement	81	0.3	1
Urban construction land	80	0.1	1
Bare land	90	0.2	1

Note: Kc is evapotranspiration coefficient of varying categories of vegetation, and the unit of the maximum root depth is mm.

**Table 5:** Area of varying sorts of RRCL in Linzhou City (unit: hm).

Type of Land	Cultivable Land			Reclaimable Land					Grand Total
	Natural Grassland	Bare Land	Total	Artificial Forest	Garden	Artificial Grassland	Pond	Total	
Area	2119.9	112.1	2233	3377.2	3208.4	8.3	18.9	6612.8	8845.8

**Table 6:** Area of RRCL in each town of Linzhou City (unit: hm).

Name of Town	Reclaimable Land	Cultivable Land	Total	Proportion
Caisang	537.50	157.00	694.50	7.85%
Chadian	489.64	165.58	655.22	7.41%
Chengjiao	335.07	71.35	406.41	4.59%
Donggang	550.64	643.82	1194.46	13.50%
Dongyao	681.13	217.44	898.57	10.16%
Guilin	487.93	281.19	769.12	8.69%
Hejian	406.54	133.41	539.95	6.10%
Heshun	597.63	549.69	1147.31	12.97%
Hengshui	576.08	1229.91	1805.98	20.42%
Linqi	483.73	33.13	516.87	5.84%
Lingyang	20.83	10.61	31.44	0.36%
Rencun	636.47	65.41	701.88	7.93%
Shibanyan	83.00	1.68	84.68	0.96%
Wulong	404.57	28.74	433.31	4.90%
Yaocun	251.15	118.42	369.57	4.18%
Yuankang	295.62	13.11	308.73	3.49%
Chengghuan	7.81	0.73	8.53	0.10%

**Table 7:** Ecosystem regulation services of RRCL of varying types of land in Linzhou City.

Type of Land		Water Conservation (mm)			Soil Retention (t·km^−2^·a^−1^)			Carbon Sequestration and Oxygen Release (gc·m^−2^)		
		Max	Mean	Min	Max	Mean	Min	Max	Mean	Min
Cultivable	Natural grassland	70.9	25.3	2.98	2476.6	101.18	0.31	311	202.6	44.45
	Bare land	452.6	288.2	116.3	1479.3	104.3	0.48	289	184.6	74.13
	Unclassified	452.6	39.5	2.98	2476.6	101.25	0.31	311.9	201.6	44.45
Reclaimable	Artificial forest	70.3	8.47	0.42	4132.2	62.4	0.22	339.2	201.4	0
	Garden	27.1	7.92	0.93	3765.5	131.1	0.23	318.5	191.8	39.6
	Artificial grassland	60.4	23.63	13.8	697.4	51.96	2.72	244.36	172.17	117.5
	Pond	0	0	0	548.3	69.76	0.28	270.3	184.3	44.2
	Unclassified	70.3	8.2	0	4132.2	95.3	0.22	339.2	196.6	0
Current cultivated land		27.6	7.07	0.93	4005.6	93.3	0.15	331.8	171.8	0

**Table 8:** Loss of ecosystem services of varying types of RRCL (unit: t).

Type of Land		Water Conservation		Soil Retention		Carbon Sequestration and Oxygen Release	
		Per Hectare	Total Loss	Per Hectare	Total Loss	Per Hectare	Overall Loss
Cultivable land	Natural grassland	182.30	386,457.77	0.08	167.05	0.31	652.93
	Bare land	2811.30	315,146.73	0.11	12.33	0.13	14.35
	Total	324.30	701,604.50	0.08	179.38	0.30	667.28
Reclaimable land	Artificial forest	14.00	47,280.80	−0.31	−1043.55	0.30	999.65
	Garden	8.50	27,271.40	0.38	1212.78	0.20	641.68
	Artificial grassland	165.60	1374.48	−0.41	−3.43	0.00	0.03
	Pond	−70.70	−1336.23	−0.24	−4.45	0.13	2.36
	Total	11.30	74,590.45	0.02	161.34	0.25	1643.72
Grand total		87.7	776,195	0.039	340.72	0.26	2311

**Table 9:** Value loss of ecological services incurred by RRCL development (unit: CNY 10,000).

Type of Land		Individual Loss			Per Hectare	Total
		Water Conservation	Soil Retention	Carbon Sequestration and Oxygen Release		
Cultivable land	Natural grassland	595.14	0.5	107.69	0.33	703.34
	Bare land	485.33	0.04	2.37	4.35	487.73
	Total	1080.47	0.54	110.06	0.53	1191.07
Reclaimable land	Artificial forest	72.81	−3.15	164.88	0.07	234.55
	Garden	42	3.66	105.84	0.05	151.5
	Artificial grassland	2.12	−0.01	0.01	0.25	2.11
	Pond	−2.06	−0.01	0.39	−0.09	−1.68
	Total	114.87	0.49	271.11	0.06	386.47
Grand total		1195.34	1.03	381.17	0.59	1577.54

## Data Availability

The data in this study for RRCL in the study period were obtained from the results of the Third National Land Resource Survey, Resource and Environment Sciences and Data Center (http://www.resdc.cn), Scientific Data Center in Cold and Arid Regions (http://westdc.westgis.ac.cn/), TOA image collection from the Landsat 8 Satellite (https://earthengine.google.com), Linzhou Fengyun Meteorological Development Center, Dataset on the Global Potential Evapotranspiration and Global Aridity Index (https://cgiarcsi.community/data/global-aridity-and-pet-database/), and territorial and spatial planning of Anyang City (accessed on 24 November 2021).
